# Is knee extension strength a key factor in badminton-specific agility among elite players?

**DOI:** 10.3389/fspor.2025.1733134

**Published:** 2026-01-02

**Authors:** Hirotaka Nakashima, Ryosuke Ando, Mai Kameda, Shinsuke Tamai, Taro Iizuka, Yoshihiro Hoshikawa, Mariko Nakamura

**Affiliations:** 1 Department of Sports Sciences, Japan Institute of Sports Sciences, Tokyo, Japan; 2 The High Performance Support Project, Japan Sport Council, Tokyo, Japan; 3 Department of Sports Science, Japan Women’s College of Physical Education, Tokyo, Japan

**Keywords:** anthropometry, lunge, quickness, racket sports, training

## Abstract

**Introduction:**

Badminton-specific agility, characterized by frequent lunges performed with the leg on the racket-holding side, is a key determinant of overall badminton performance. Although leg extension strength is expected to play a significant role, the factors that influencing badminton-specific agility remain unclear. This study therefore aimed to test the hypothesis that knee extension strength is correlated with badminton-specific agility in world-class and elite/international-level badminton players.

**Methods:**

This study included twenty-seven male and twenty-three female professional badminton players from the Japanese national team. Participants completed two tests: (1) a badminton-specific agility test measuring the time required to reach sensors at the four corners of a singles court using badminton-specific movement, and (2) an isokinetic knee extension strength test at angular velocities of 60 °/s and 180 °/s. The Spearman rank-order correlation coefficients were used to assess the relationships between them (*P* < .05).

**Results and discussion:**

Significant inverse correlations were found between knee extension torque normalized to body mass for the leg opposite the racket-holding hand and badminton-specific agility at both 60 °/s and 180 °/s (males at 60 °/s: *r_s_* = −.619; 180 °/s: *r_s_* = −.579; females at 60 °/s: *r_s_* = −.445; 180 °/s: *r_s_* = −.446). In contrast, only the same-side leg at 60 °/s showed a significant inverse correlation (males: *r_s_* = −.413; females: *r_s_* = −.490). Overall, these results show that knee extension strength is crucial for badminton-specific agility among world-class and elite/international-level male and female badminton players. Furthermore, our findings suggest differing demands for force production between the legs on the same and opposite sides of the racket-holding hand.

## 1 Introduction

Badminton is a fast-paced racket sport, in which players hit a shuttlecock in rallies occurring approximately once per second (1, 2). During these high-tempo rallies, players require to cover the entire court (5.18 m × 6.70 m for singles and 6.10 m × 6.70 m for doubles) to respond to diverse opponent shots. Therefore, agility plays an important role in badminton.

Agility in badminton is uniquely characterized by movements within a confined space and frequent lunges, forward, backward, and sideward, with the same leg as the racket-holding hand, accounting for 15%–20% of all movements (3, 4). These movements facilitate rapid transitions to optimal positions for the shot, followed by quick returns to the starting position or seamless direction changes for subsequent actions (4, 5). This specialized form of agility, known as “badminton-specific agility,” is a key determinant of overall badminton performance. Indeed, previous studies have shown that players with higher performance levels tend to exhibit greater badminton-specific agility (6, 7). Therefore, it is essential for badminton players to enhance their badminton-specific agility to improve their overall performance.

However, the determinants of badminton-specific agility remain unclear. Sheppard and Young (8) previously defined agility as comprising change-of-direction speed and perceptual/decision-making factors. The physical component of change-of-direction speed can be further divided into the straight sprinting speed, leg muscle quality (defined in this study as leg muscle strength), anthropometry, and technique. Given the limited court space, straight sprinting speed is unlikely to be relevant; indeed, Madsen et al. (7) found no significant correlation between the straight sprinting speed and badminton-specific agility in elite players. Thus, excluding perceptual/decision making factors and technical aspects, the primary contributors appear to be leg muscle strength and anthropometry, which are key considerations for strength and conditioning practice.

Few studies have directly examined the relationship between leg muscle strength, anthropometry, and badminton-specific agility. In related studies, Lam et al. (9) reported that male players demonstrated greater lunge speed than females, attributing this difference to superior muscular strength. Furthermore, Guan et al. (10) also showed that lunge speed during fencing, a sport involving similar frequent lunges, was correlated with knee extension torque of the back leg (equivalent to the opposite the racket-holding hand in badminton) during lunging. Taken together, we hypothesized that badminton-specific agility would be associated with the knee extension strength of the leg opposite the racket-holding hand. From an anthropometric perspective, players with a greater lean body mass and lower fat mass are theoretically expected to exhibit superior agility. However, the extent to which these hypotheses are applicable to elite badminton players remains unclear.

Therefore, the present study aimed to examine the relationships among badminton-specific agility, knee extension strength, and anthropometry in elite male and female badminton players.

## 2 Materials and methods

### 2.1 Experimental design and ethical statements

This study employed a cross-sectional experimental design to address the aforementioned aims. Elite badminton players completed the following assessments: (1) badminton-specific agility test, (2) isokinetic concentric knee extension strength test, and (3) anthropometric measurements. For completeness, the assessments for (2) and (3) were conducted in accordance with the guidelines detailed in the handbook on athlete physical fitness testing (11). All assessments were conducted on the same day, following adequate warm-up, familiarization, and rest. The relationships among these values were examined.

This study was approved by the Ethics Committee of the Japan Institute of Sports Sciences (No. 2025-007) and was conducted in accordance with the Declaration of Helsinki. Written informed consent was obtained from all participants prior to data collection. Furthermore, we disclosed the study details on the institution's website and provided an opt-out option, allowing participants to withdraw their consent for data.

### 2.2 Participants

We conducted an *a priori* power analysis using the point biserial correlation model in G*Power 3.1.9.7 to justify the sample size (12). We set the effect size at 0.5, two-tailed 0.05, and the power at 0.8. The power analysis showed that a minimum of 26 participants for each sex was required. Thus, 31 male and 29 female badminton players who agreed to participate were recruited. However, some participants withdrew from the study shortly before the experiment due to injuries. Consequently, 27 male and 23 female badminton players participated in the study (age: males 24.0 ± 3.1 years; females 23.2 ± 2.5 years). All participants were members of the Japanese national badminton team and were classified as tier 5 (world class) or tier 4 (elite/international-level), based on the criteria proposed by McKay et al. (13). Although the sample size for female players did not meet the required threshold, we did not recruit additional participants due to the lack of comparably skilled players in the country.

### 2.3 Badminton-specific agility test

**Figure 1:**
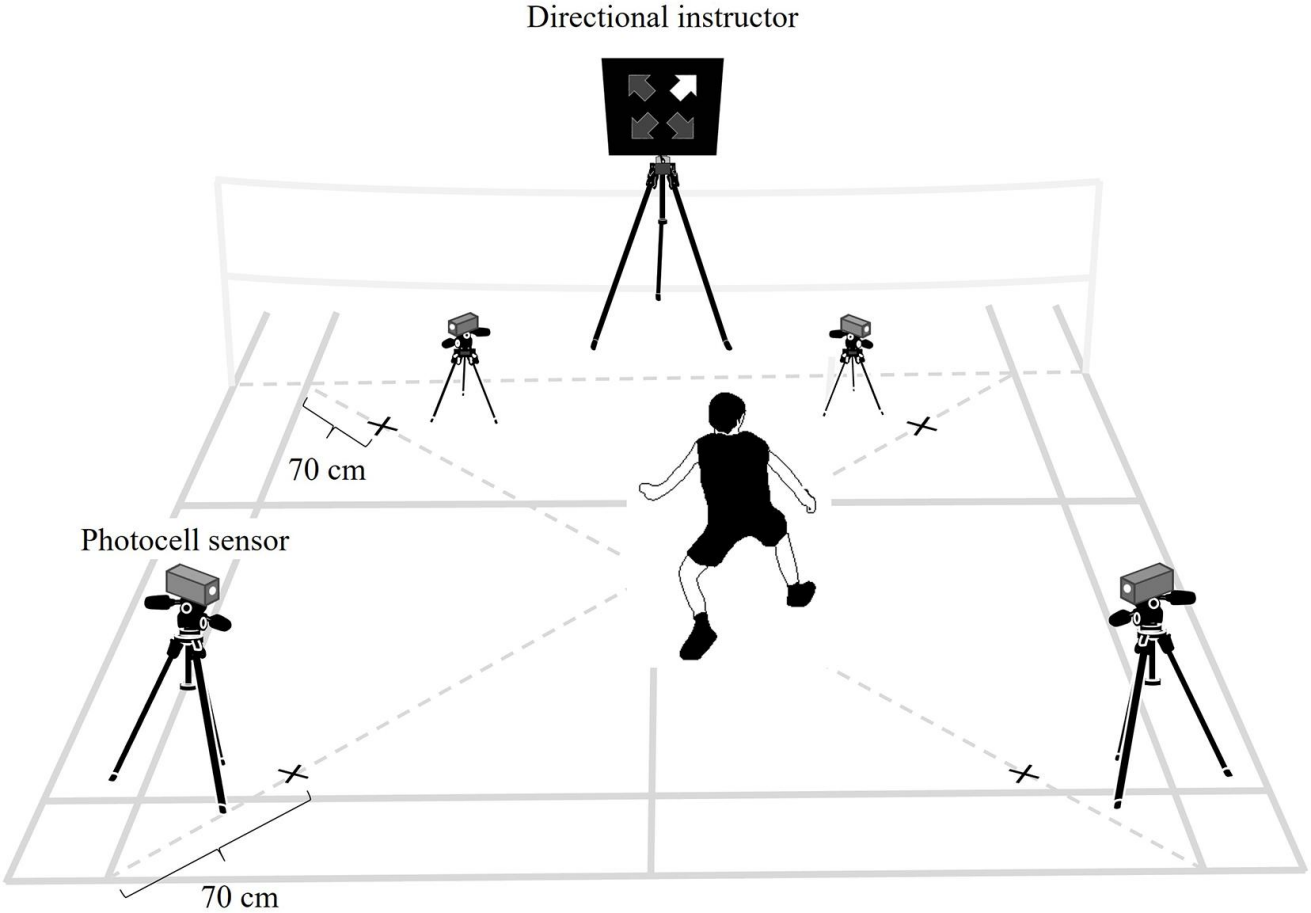
Experimental setting for the badminton-specific agility test.

We used a modified version of the test developed by Ando et al. (6) to assess badminton-specific agility. The test was conducted on a simulated badminton court. We replicated the environmental setting as described by Ando et al. (6). We placed four photocell sensors (Assist Co. Ltd., Tokyo, Japan) at each corner of a single badminton court and installed a directional indicator on the net at the center of the court (Figure 1). The front sensors were positioned at knee height, and the back sensors at elbow height.

The participants began the test at the center of the court and moved toward the sensor designated by the directional indicator. They were instructed to move as quickly as possible using badminton-specific footwork, and reach for the sensor with their dominant hand. Subsequently, they returned to the center and moved toward the next sensor as directed. The subsequent directional cue appeared 1.0 s following the previous sensor detected the participant's arrival. We determined the cue interval by simulating the average tempo of single matches among elite badminton players (1, 2). Each set comprised eight cues, and the order of directional cues was randomized. The participants completed six sets, with 20 s of rest between each set.

We recorded the time taken to reach the designated sensor from the moment the directional cue was displayed, which was automatically measured using a sensor system synchronized with the cue presentation. We excluded the final trials in each set (eighth direction), which could be predictable, as well as trials for which the sensor was not cleanly activated, and those in which the participant moved in the wrong direction. We calculated the average of these reach times (hereafter “reach time”) to serve as the parameter for badminton-specific agility.

### 2.4 Isokinetic concentric knee extension strength test

We assessed knee extension strength based on the isokinetic concentric knee extension torque using an isokinetic dynamometer (Biodex System 4; SAKAI Medical Co., Ltd., Tokyo, Japan). Participants were seated with their hips flexed at 85° from the standard anatomical position, and stabilization straps were applied across the trunk, waist, and thigh of the measurement leg to minimize compensatory movements. The seat was adjusted to ensure alignment between the knee joint and the dynamometer's rotational center, and the participant's leg was secured to the dynamometer using padded straps. The participants performed three consecutive concentric knee extensions, at an angular velocity of 180 °/*s* and two consecutive knee extensions at 60 °/*s*. The range of motion for knee extension was approximately 90°–180°. This protocol was repeated in the contralateral leg. The peak torques at each angular velocity for both legs were normalized to the body mass (Nm/kg) and used as parameters for knee extension strength. Knee extension strength was measured at two different angular velocities because force production speed varies throughout the lunge, and these measurements assess muscle function across that range.

### 2.5 Anthropometric measurements

We measured the body height using a digital stadiometer (AD-6228A; A&D Company Ltd., Tokyo, Japan). Body mass, body fat mass, and lean body mass were measured using a calibrated digital scale and an air displacement plethysmograph (BODPOD; Cosmed Srl, Roma, Italy). Participants wore swimsuits and swim caps to minimize the air trapped in their clothing and hair, which could affect the measurements. After weighing on the scale, the participants entered the chamber to measure their body and thoracic gas volumes. Body fat mass was calculated using the equation reported by Brozek et al. (14) Lean body mass was calculated as body mass minus body fat mass.

### 2.6 Statistical analysis

Statistical analyses were conducted using SPSS statistical software (version 24.0, IBM, NY, USA). Normality was tested using the Shapiro–Wilk test, which indicated some parameters were not normally distributed. Therefore, the Spearman rank-order correlation coefficients were used to assess the relationships between the reach time of badminton-specific agility and knee extension torque normalized to body mass and anthropometric variables, stratified by sex. The statistical significance level was set at *P* < .05.

## 3 Results

**Figure 2:**
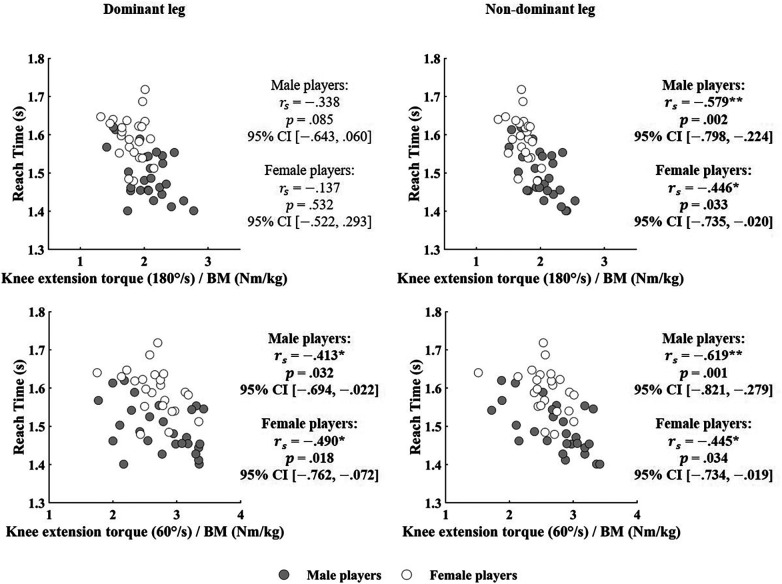
Relationship between reach time of the badminton-specific agility and knee extension torque normalized to body mass.

**Figure 3:**
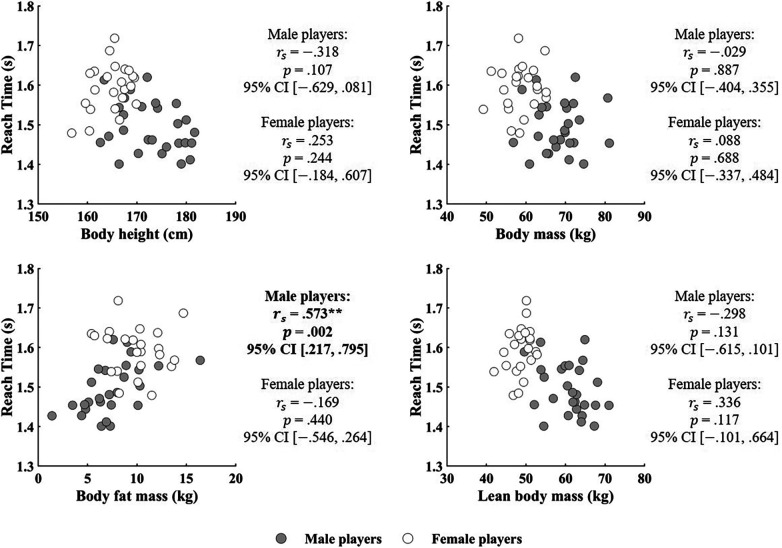
Relationship between reach time of the badminton-specific agility and anthropometric variables.

Table 1 summarizes the reach times and knee extension torque normalized to body mass, while Table 2 summarizes the anthropometric variables. Significant inverse correlations were observed between knee extension torque at both 180 °/s and 60 °/s normalized to the body mass and reach time for the non-dominant leg in both male and female players (Figure 2). In contrast, significant inverse correlations were found between knee extension torque at 60 °/s normalized to body mass and reach time for the dominant leg in both male and female players, whereas no significant correlation was observed at 180 °/s (Figure 2). A significant correlation was observed between body fat mass and reach time in male players, whereas no significant correlations were identified for the other anthropometric variables (Figure 3).

**Table 1:** Mean (SD), maximum, and minimum reach time of badminton-specific agility test and knee extension torque normalized to body mass.

Sex	Descriptive statistics	Reach time of badminton-specific agility test (s)	Knee extension torque normalized to body mass (Nm/kg)			
			Dominant leg		Non-dominant leg	
			180 °/s	60 °/s	180 °/s	60 °/s
Male	Mean (SD)	1.49 (0.06)	2.07 (0.33)	2.77 (0.52)	2.03 (0.27)	2.73 (0.49)
	Maximum	1.62	2.77	3.42	2.54	3.42
	Minimum	1.40	1.41	1.77	1.51	1.72
Female	Mean (SD)	1.59 (0.06)	1.80 (0.21)	2.66 (0.36)	1.72 (0.16)	2.56 (0.32)
	Maximum	1.72	2.15	3.34	2.02	3.01
	Minimum	1.48	1.32	1.75	1.34	1.51

The dominant and non-dominant legs are the same and opposite legs from the racket-holding hand, respectively.

**Table 2:** Mean (SD), maximum, and minimum anthropometric variables.

Sex	Descriptive statistics	Body height (cm)	Body mass (kg)	Body fat mass (kg)	Lean body mass (kg)
Male	Mean (SD)	172.9 (6.0)	68.4 (5.8)	7.4 (3.0)	61.0 (5.3)
	Maximum	181.7	81.1	16.4	71.0
	Minimum	162.6	56.8	1.4	49.6
Female	Mean (SD)	164.9 (3.7)	58.6 (4.3)	9.9 (2.5)	48.7 (2.7)
	Maximum	169.9	65.1	14.7	52.8
	Minimum	156.8	49.2	5.4	41.9

## 4 Discussion

The main findings of this study were as follows: 1) knee extension strength of the non-dominant leg was associated with reach time; 2) knee extension strength of the dominant leg at a lower angular velocity was associated with reach time; and 3) body fat mass was associated with reach time in male players. Although previous study on collegiate badminton players has shown that lower-limb strength influences performance in the side-shuffle test, which is a representative agility test widely used in Japan (15), the present study extends this evidence to world-class or elite/international-level athletes. These findings highlight that fundamental physical qualities remain important even among elite players.

With respect to the non-dominant leg, significant inverse correlations were identified between knee extension torque normalized to body mass and reach time. Badminton involves frequent lunge movements (3, 4). Similar to a previous study on fencing (10), in badminton, the knee extension strength of the non-dominant leg would contribute to increased lunge speed, thereby enhancing badminton-specific agility. Although previous studies have demonstrated a relationship between knee extension torque and a single-lunge speed (10), this study extends this evidence to a more complex, sport-specific agility task that integrates technical and perceptual demands. These findings suggest that, despite the multifactorial nature of agility, fundamental lower-limb strength remains critical for reducing movement time. In addition, significant inverse correlations were observed at high (180 °/*s*) and low (60 °/*s*) angular velocities. Badminton-specific agility involves not only accelerating from a stationary position (i.e., the first instruction of each set) but also from a moving condition (i.e., the second to eighth instruction of each set). Therefore, improving knee extension strength of the non-dominant leg at each speed range may enhance badminton-specific agility.

For the dominant leg, only knee extension torque at 60 °/*s* normalized to body mass inversely correlated with reach time, whereas no such correlation was observed at 180 °/*s*. This is likely related to the functional role of the dominant leg during lunging, which involves decelerating through eccentric contraction and subsequently returning to the initial position through concentric contraction (9, 16). The relatively long foot contact time during these actions (approximately 0.6–0.7 s) indicates that slower force generation is particularly relevant (17, 18). Thus, strength at lower angular velocities appears essential for efficient recovery from lunges and improved agility performance.

Regarding anthropometry, body fat mass was found to be negatively correlated with reach time in male players. From a biomechanical perspective, increased body fat adds to body mass without contributing to force production, thereby impairing acceleration and deceleration capacities. Conversely, no such correlation was found in female players. One possible explanation for the lack of a significant correlation in female players is that the standard deviation of body fat mass in female players was smaller than that in male players.

It is worth noting the trainability of elite badminton players. According to a handbook reporting on the physical fitness of elite Japanese athletes (11), fencing players exhibited greater knee extension strength than badminton players in this study (average knee extension strength for fencing players were as follows: 2.33 Nm/kg for males at 180 °/*s*; 3.13 Nm/kg for males at 60 °/*s*; 1.92 Nm/kg for females at 180 °/*s*; and 2.80 Nm/kg for females at 60 °/*s*). All participants in this study were highly trained, and the measured values may serve as benchmarks for other players; however, there may still be room for improvement in their knee extension strength. Thus, this study suggests that even elite players can improve their knee extension strength and potentially enhance their badminton-specific agility. Strength and conditioning professionals should therefore consider incorporating targeted lower-limb training to optimize agility in elite badminton players. Specifically, strengthening the non-dominant knee extensor muscles, which contribute to increased lunge speed, may enhance badminton-specific agility. Performing lunges from various states, such as a stationary position and a condition with a certain amount of speed, during a badminton match, training of the non-dominant knee extensor muscles from the low-to-high-speed range is essential. Additionally, strengthening the dominant knee extensor muscles reduces the time needed to return to the original position after lunging. The slow nature of this return movement suggests that training the dominant knee extensor muscles in the low-speed range would be effective. In this context, training in low- and high-speed ranges refers to movement velocity, as described below. For example, in a leg extension exercise with a knee joint range of motion of 90°, training in the low-speed range involves performing the extension over 1.5 s (60 °/*s*), whereas training in the high-speed range requires completing the extension in 0.5 s (180 °/*s*). In summary, training the knee extensor muscles at appropriate speed range corresponding to lunge movements may enhance badminton-specific agility.

Furthermore, the findings of this study could be applied to other sports that require frequent lunges and agility within confined spaces, such as tennis and squash. Wilkinson et al. (19) emphasized that agility plays a critical role in the success of elite squash players. The rally tempo in squash is also rapid, typically only 1.0–1.5 s (20), and players are required to return to the center position after each shot. Given the similarities between squash and badminton, we hypothesized that knee extension strength is a significant factor in squash agility.

## 5 Conclusion

This study investigated the relationships among badminton-specific agility, knee extension strength, and anthropometric characteristics in elite male and female badminton players. This study showed that knee extension strength is one of the important determinants of badminton-specific agility among elite players. Moreover, our findings indicate differences in force production requirements between dominant and non-dominant legs for badminton-specific agility.

## Data Availability

The dataset used in this study is not publicly available due to ethical restrictions. Data are available from the corresponding author upon reasonable request. Requests to access the datasets should be directed to Hirotaka Nakashima, hirotaka.nakashima@jpnsport.go.jp.
